# Editorial: Phosphodiesterases as Drug Targets in Airway and Inflammatory Diseases

**DOI:** 10.3389/fphar.2021.657596

**Published:** 2021-04-12

**Authors:** Juraj Mokry, Mark Giembycz, Daniela Mokra

**Affiliations:** ^1^ Department of Pharmacology, Jessenius Faculty of Medicine, Comenius University, Martin, Slovakia; ^2^ Department of Physiology and Pharmacology, University of Calgary, Calgary, AB, Canada; ^3^ Department of Physiology, Jessenius Faculty of Medicine, Comenius University, Martin, Slovakia

**Keywords:** Phosphodiesterase, PDE inhibitor, roflumilast, Respiratory disease, Ciliary activity

Recently, several new drugs have been introduced to treat obstructive lung diseases associated with inflammation (e.g., bronchial asthma, chronic obstructive pulmonary disease [COPD]), including long-acting bronchodilators, corticosteroids and monoclonal antibodies. Despite these new medicines, there are still patients who might benefit from a more selective approach to treatment given that this may be accompanied by fewer adverse effects, especially when a combination of drugs is required to manage severe disease. The possibility that inhibiting certain phosphodiesterase (PDE) isoenzymes (in particular PDE3 and PDE4) and, thereby, increasing the concentration of cyclic adenosine monophosphate (cAMP) in specific tissues and organs, may have therapeutic benefit has gained general acceptance over the last 30 years. Indeed, PDEs have become attractive drug targets because an increase in cAMP can relax smooth muscle and suppress inflammation.

The articles in this series of mini-reviews focus on the involvement of various PDE isoforms in the mechanisms underlying inflammatory and other airway diseases. As described by Beute et al., PDE3 plays a critical role in basophil and mast cell degranulation and, therefore, its inhibition by a small molecule inhibitors such as enoximone may be a viable treatment option in allergic airway inflammation. Similarly, another selective PDE3 inhibitor RPL554 (ensifentrine) has been intensively tested during last years and exhibits both bronchodilator and anti-inflammatory activity in *vitro* and *in vivo* assays. The benefits of inhaled ensifentrine in clinical studies with asthma and COPD patients is reviewed by Phillips and showed predominantly bronchodilator effects without reliable evidence for anti-inflammatory activity usually associated with PDE4 inhibition (Cazzola et al., 2019). This can be explained by moderately potent PDE3 inhibition but only weakly potent PDE4 inhibition of this agent, as described by Boswell-Smith et al. (2006).

Currently, roflumilast is the only selective PDE4 inhibitor that has been approved for the treatment of a particular subset of patients with COPD. Disease guidelines recommend that roflumilast be used as an add-on therapy in people categorized as high risk, having severe, symptomatic COPD in whom exacerbations occur despite regular treatment with a combination of a long-acting β_2_-adrenoceptor agonist (LABA), a long-acting muscarinic receptor antagonist and an inhaled corticosteroid. However, roflumilast has a low therapeutic ratio and ways to improve effectiveness and tolerability are needed. Possibilities include the development of dual- or multi-specificity agents. Another potential approach to improve the tolerability and therapeutic index is to develop PDE4 inhibitors for inhaled administration. Several examples exist and some have advanced to clinical trials for the treatment of asthma and COPD. Phillips, in his brief update, summarizes the pharmacological and clinical details of several inhaled PDE4 inhibitors (e.g. CHF6001) as well as those which failed to demonstrate sufficient efficacy in clinical trials. With this in mind, Stellari et al. have described the anti-inflammatory activity of the novel PDE4 inhibitor, CHF6001, showing specifically that topical administration efficiently disrupts NF-κB activation associated with lipopolysaccharide challenge, an effect that may be relevant to the prevention of exacerbations in patients with COPD.

The articles by Kawamatawong and Janosova et al., review the potential use of selective PDE4 inhibitors in non-COPD respiratory diseases, providing evidence of their efficacy in controlling symptoms in allergic rhinitis/rhinosinusitis and diseases associated with mucus hypersecretion. However, few PDE4 inhibitors have progressed to phase II and III clinical trials for these indications because of weak clinical efficacy and adverse effects. The treatment of respiratory diseases associated with non-Th2 (e.g., neutrophilic) inflammation such as non-Th2 asthma, asthma-COPD overlap, and non-Th2 allergic rhinitis represent a clear, unmet clinical need for which PDE4 inhibitors could be effective.

More light into the mechanisms governing ciliary motility and mucociliary clearance in respiratory disorders and the involvement of PDE is provided by Joskova et al.. In their review, they conclude that selective or dual PDE inhibitors may represent important tools in supporting normal cilia function and effective mucokinetic activity. For example, PDE1A inhibitors potentiate the cilia-stimulatory effects of β_2_-adrenoceptor agonists and this is mediated by cAMP; in contrast, PDE4 inhibitors become effective in the presence of LABAs to stimulate ciliary beating. This makes the mucociliary clearance in pathological airway conditions more effective. Furthermore, dual PDE3/4 inhibitors could provide additional benefits and show a more rapid response when taken with β_2_-adrenoceptor agonists; however, their cilia-stimulatory effect is probably mediated predominantly by PDE4 inhibition. The role of PDE inhibitors in airway cilia-driven transport may help in prevention of progressive loss of pulmonary function often observed despite current therapy.

Despite the fact that PDE inhibitors have been studied for decades, their full therapeutic potential in respiratory diseases after systemic and local (inhaled) administration is still unclear. Therefore, a continued effort to collect sufficient evidence about the efficacy and safety of these molecules in animal models and in the clinical setting is essential.
